# An Experimental Study of Dislocation Dynamics in GaN

**DOI:** 10.3390/mi14061190

**Published:** 2023-06-02

**Authors:** Eugene B. Yakimov, Yury O. Kulanchikov, Pavel S. Vergeles

**Affiliations:** Institute of Microelectronics Technology RAS, Chernogolovka 142432, Russia; kulanchikov@iptm.ru (Y.O.K.); vergeles@iptm.ru (P.S.V.)

**Keywords:** dislocation mobility, GaN, indentation, cathodoluminescence, EBIC, low-energy electron beam irradiation

## Abstract

The dynamics of dislocations introduced through indentation or scratching at room temperature into a few GaN layers that were grown using the HVPE, MOCVD and ELOG methods and had different dislocation densities were studied via the electron-beam-induced current and cathodoluminescence methods. The effects of thermal annealing and electron beam irradiation on dislocation generation and multiplication were investigated. It is shown that the Peierls barrier for dislocation glide in GaN is essentially lower than 1 eV; thus, it is mobile even at room temperature. It is shown that the mobility of a dislocation in the state-of-the-art GaN is not entirely determined by its intrinsic properties. Rather, two mechanisms may work simultaneously: overcoming the Peierls barrier and overcoming localized obstacles. The role of threading dislocations as effective obstacles for basal plane dislocation glide is demonstrated. It is shown that under low-energy electron beam irradiation, the activation energy for the dislocation glide decreases to a few tens of meV. Therefore, under e-beam irradiation, the dislocation movement is mainly controlled by overcoming localized obstacles.

## 1. Introduction

GaN and GaN-based heterostructures are now widely used in the fabrication of optoelectronic, radio frequency and high-power electronic devices. Consequently, most research on this compound has focused on exploring its electrical and optoelectronic characteristics. Dislocation studies have received little attention, despite the fact that dislocations can be introduced into GaN at room temperature [1,2,3,4,5,6,7,8,9,10,11]. Dislocations are one-dimensional defects which essentially affect the mechanical properties of crystalline materials [12,13]. Dislocation effects on the electrical and optical properties of semiconductor materials are also well documented [13,14,15,16]. They can also affect other physical properties, such as thermal conductivity [17] or resistive switching [18]. The dislocation effects on the macroscopic electrical and optical properties of the state-of-the-art GaN seem to be less significant than that observed in other semiconductors [19,20,21,22], although it has been already shown that they noticeably increase the local recombination rate [23,24,25], lead to Mg redistribution [26], affect the quality of p-n junctions [27,28] and high-electron-mobility transistors [29,30,31] and can enhance the degradation of light-emitting and laser diodes [32]. As shown in [33], the decrease in threading dislocation density can lead to the increase in photoluminescence intensity. Recently, it was shown that dislocations can convert the conductivity type from n- to p-type conductivity [34]. Thus, the reliable prediction of the performance of devices based on GaN requires a better understanding of their mechanical characteristics, in addition to their optical and electrical performance, since loading during processing or packaging may introduce dislocations that can significantly degrade the performance of these devices. Knowledge of the structure of dislocations induced by processing can lead to the development of low-cost and low-dislocation-density GaN wafers. Different processing steps, such as wafer sawing and grinding, thermal stresses, chemical–mechanical polishing, film–substrate mismatch, etc., can also lead to the appearance of stress, which can suppress the processing yield and application reliability of microelectronic devices. Moreover, as shown in [35], even the type of carrier gas used during growth can affect GaN mechanical properties. The ability of dislocations in GaN to move at room temperature under mechanical stress has been demonstrated using many methods, such as chemical etching [36], transmission electron microscopy [1,2,3,4,5,36,37,38,39], cathodoluminescence (CL) and electron-beam-induced current (EBIC) methods [1,2,3,4,5,6,7,8,9,10,11,37,38,39]. However, to date, the overwhelming number of studies on GaN mechanical properties have been carried out using indentation, through which the hardness, Young’s modulus and yield stress were obtained. The dynamics of dislocations, particularly their mobility and the activation energy for their glide, have not been studied in practical terms. Only a few experimental and theoretical investigations in which the dislocation mobility of GaN was considered can be mentioned [8,10,11,40,41,42,43,44,45]. As a wide-bandgap semiconductor, GaN is a suitable material for devices operating at elevated temperatures. To predict dislocation behavior in such devices, the activation energy for the dislocation mobility *E_d_* must be known. In [46,47], *E_d_* was estimated using its empirical correlation with the bandgap value, and a value of approximately 2 eV was obtained. Estimations using the empirical correlation between *E_d_* and the *Gb*^3^ value, where *G* is the shear modulus and *b* is the Burgers vector of dislocation, gave approximately the same value [48]. However, the ability of dislocations to move at room temperature implies that the activation energy for dislocation mobility in GaN is well below 1 eV, which has been confirmed via both molecular dynamics simulations [43] and experimental estimations [8,10] based on measurements of dislocation travelling distances from the indentation as a function of temperature. It is widely accepted that for dislocation glide in semiconducting covalent crystals, an intrinsic potential barrier, the so-called Peierls potential, must be overcome and that this barrier determines the dislocation mobility. Overcoming the Peierls barrier is achieved through two successive processes: the formation of double kinks on a straight dislocation line and the subsequent migration of the generated kinks along the dislocation. In all previous papers, dislocation transport in GaN was discussed in the framework of such a model. However, if the Peierls barrier is low, how does this occur in GaN, some additional mechanisms may essentially affect dislocation mobility, contrary to long-held assumptions. For example, if the material contains some obstacles to dislocation motion, two mechanisms may work simultaneously: overcoming the Peierls barrier and overcoming localized obstacles. Thus, the net dislocation velocity will be controlled by both the waiting time of the dislocation pinned by obstacles and the travelling time between the stable configurations determined by the double-kink mechanism [49]. At high temperatures corresponding to low stresses, deformation is controlled by the localized obstacles, and the activation energy should be higher than that of the double-kink mechanism [49]. Possibly, this explains the large difference between activation energies estimated at high temperatures [50] and those estimated at temperatures near room temperature [8,10]. It should be emphasized that such dislocation behavior is relatively unique, since in most semiconducting covalent crystals, dislocation mobility is mainly controlled by the double-kink mechanism. It should also be mentioned that in our previous works [8,10], differences in the activation energies for dislocation glide in n- and p-GaN were revealed. The dopant effect on activation energy in other semiconductor crystals is well known [51,52]. Moreover, in some materials, donors and acceptors demonstrate the opposite effect [51]. Therefore, it would be interesting to examine the dopant effect in GaN more carefully. It was also shown that low-energy electron beam irradiation (LEEBI) enhances the dislocation mobility (the so-called recombination-enhanced dislocation glide (REDG)) in GaN [7,11,38,53,54,55]. As shown in [38], some dislocations can move under electron beam excitation even at liquid nitrogen temperature, which means that, in such conditions, the activation energy for dislocation glide is close to 0. It is similar to REDG in 4H-SiC, for which it was shown that the low activation energy for REDG is determined by the very low activation energy for kink migration along partial dislocations [56,57]. It should be noted that, contrary to the case of 4H-SiC, in all measurements of REDG in GaN, only a small number of dislocations became mobile, which allows one to assume that dislocation pinning on obstacles essentially affects the dislocation motion. Thus, it seems that to describe the dislocation dynamics in GaN, the effects of both the Peierls barrier and obstacles should be estimated. As the number and the strength of obstacles should depend on the growth method and the defect concentration, for this purpose, it is useful to compare dislocation dynamics in different GaN layers.

In the present study, investigations of the thermal annealing effect and LEEBI on the motion of dislocations introduced into a few GaN epilayers at room temperature were carried out. In addition to offering a deeper understanding of the obstacle effect on dislocation motion and multiplication, such experiments imitate the dislocation behavior during the operation of devices based on GaN at elevated temperatures.

## 2. Materials and Methods

A few GaN samples with different dopant concentrations and dislocation densities were used in the experiments: S1—400 μm thick n-GaN grown using hydride vapor phase epitaxy (HVPE) from Kyma Technolgies Inc. (Raleigh, NC, USA) with the donor concentration of (4–5) × 10^16^ cm^−3^ and a dislocation density of approximately 10^7^ cm^−2^; S2—3 μm thick n-GaN grown via metalorganic vapor-phase epitaxy (MOCVD) with the donor concentration of 10^17^ cm^−3^ and a dislocation density of approximately 10^9^ cm^−2^; S3—4 μm thick n-GaN grown via MOCVD with the donor concentration of 2 × 10^17^ cm^−3^ and a dislocation density of approximately 10^8^ cm^−2^; S4—1.6 μm thick p-GaN grown via MOCVD with the Mg concentration of 5 × 10^19^ cm^−3^ and a dislocation density exceeding 10^8^ cm^−2^, S5—6 μm thick n-GaN grown via epitaxial lateral overgrowth (ELOG) with the donor concentration ~10^17^ cm^−3^ and dislocation densities of approximately 10^8^ cm^−2^ above the windows in the SiO_2_ mask and ~10^6^ cm^−2^ in the wings (more details can be found in [58]); and S6—60 μm thick n-GaN grown via HVPE with the donor concentration of 10^17^ cm^−3^ and a dislocation density of approximately 10^8^ cm^−2^. In all samples studied, the dopant concentration was obtained using capacitance–voltage measurements and the grown-in dislocation density, determined via the electron-beam-induced current (EBIC) method.

The samples were plastically deformed at room temperature through indentation with a Vickers or conical indenter in an as-grown basal {0001} plane under loads between of 0.2 and 1 N or scratching with a diamond scriber that allowed for the use of small-sized samples. Deformation-induced dislocations were revealed using the EBIC or cathodolumiscence (CL) method. CL investigations were carried out using a JSM 6490 scanning electron microscope (SEM) (Jeol, Tokyo, Japan) equipped with a MonoCL3 (Gatan, Abingdon, UK) system with a Hamamatsu photomultiplier as a detector. The CL images were obtained at room temperature in the panchromatic mode or in the monochromatic mode at a wavelength of 365 nm (near-bandgap emission) or 400 nm. As shown in [6,7,9,38,39,59,60], emission at 400 nm (3.1 eV) is associated with dislocation-related luminescence (DRL). This DRL was explained in [6,38,39] using the dissociated screw dislocations in the basal plane. However, its low thermal stability and fast disappearance under electron beam irradiation allowed to assume that it is more likely determined by point defects created by moving dislocations [7,60], similar to dislocation trails observed in Si [61,62,63]. In most cases, CL measurements were carried out with the *E_b_* of 10 keV and *I_b_* in the range from 0.1 to 1 nA. At such a beam energy, the electron penetration depth is approximately 420 nm and the depth dependence of CL emission has a maximum of approximately 80 nm.

The EBIC measurements were carried out using the JSM-840 SEM (Jeol, Japan) at room temperature with a beam energy *E_b_* of 25 or 35 keV, which provides a better spatial resolution due to the submicron diffusion length of GaN [19,24,64], and a beam current of 10^−10^ A. A Keithley 428 current amplifier was used in the EBIC measurements. The Schottky diodes for the EBIC measurements were prepared via the vacuum evaporation of Au through a shadow mask with a typical diode diameter of 1 mm. Ohmic contacts were formed via In soldering to the sample. LEEBI was carried out with the same microscopes JSM-840 and JSM 6490.

As dislocations are introduced via indentation or scratching, it is useful to understand what information can be extracted from measurements of dislocation travelling distance. The dependence of the dislocation velocity on stress and temperature in covalent semiconductors is usually described using the empirical formula [13,51]:

(1) $$V=V_{0}{\left(\frac{\tau }{\tau_{0}}\right)}^{m}\exp(-\Delta E/kT),$$

where the constants *m*, *V*_0_ and τ_0_ are determined experimentally for particular materials, and Δ*E* is the activation energy for dislocation mobility. Of course, *V*_0_, *m* and τ_0_ differ for different dislocation types and different slip planes. Moreover, in a typical case, *m* depends on stress and temperature [13]. However, for estimations, the difference can be neglected, especially taking into account the large differences between the results of different works. Thus, for τ_0_, a value of approximately 1 MPa was assumed [47], while the simulation described in [65] gave values of 45–50 MPa, 10^3^ m/s and 3–4 for τ_0_, *V*_0_ and *m*, respectively, for the prismatic slip plane. However, a value exceeding 2 eV was obtained in [65] for Δ*E*, which, as noted above, contradicts the observations of dislocation mobility at room temperature. In [43], molecular dynamics simulation gave values well below 1 eV for Δ*E* and approximately 5 for *m* at temperatures close to room temperature. Despite this wide scattering, these values give us an idea of the order of magnitude of *V*_0_, *m* and τ_0_. If one takes into account the fact that τ decays with the distance from the imprint center *r* and that this decay can be described as τ = *AP*/*r*^2^, where *A* is the parameter depending on Poisson’s ratio and *P* is the applied load [66], Equation (1) can be rewritten as [8,10,66]:

(2) $$V=\frac{dr}{dt}=V_{0}{\left(\frac{AP}{\tau_{0}r^{2}}\right)}^{m}\exp(-\Delta E/kT).$$

After integration,

(3) $$\begin{array}{l} \int_{0}^{L}r^{2m}dr=L^{2m+1}=\int_{0}^{t_{d}}V_{0}{\left(\frac{AP}{\tau_{0}}\right)}^{m}\exp(-\Delta E/kT)=V_{0}{\left(\frac{AP}{\tau_{0}}\right)}^{m}t_{d}\exp(-\Delta E/kT)\\ L=V_{0}^{1/(2m+1)}{\left(\frac{AP}{\tau_{0}}\right)}^{m/(2m+1)}t_{d}^{1/(2m+1)}\exp[-\frac{\Delta E}{kT(2m+1)}] \end{array},$$

where *L* is the travelling distance of the leading dislocations measured from the imprint center, and *t_d_* is the deformation dwell time. These expressions allow us to understand how the size of dislocation rosettes depends on the load and temperature, if the dislocation mobility is determined by the double-kink mechanism.

## 3. Results

**Figure 1:**
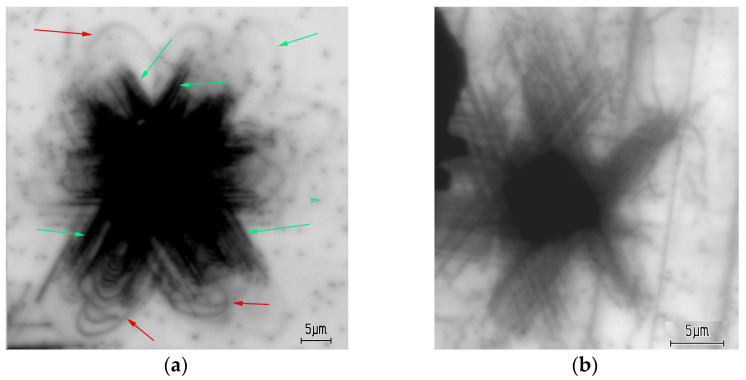
Typical EBIC images of indentations in S1 (**a**) and S5 samples (**b**) created at *E_b_* = 25 keV. Dislocations gliding in the basal plane and in those intersection the surface are shown with red and green arrows, respectively.

A typical EBIC image of a room-temperature imprint in the S1 sample, created with a load of 0.4 N, is shown in Figure 1a. As dislocations enhance the local recombination rate, they are revealed as dark lines or points in the EBIC mode. It can be seen that dislocations induced via indentation are arranged in a rosette shape with six dislocation arms running in the <11 $\bar{2}$ 0> directions along several slip systems (shown with green arrows), which correlates well with the images observed via EBIC in [7] and via CL in [1,2,3,4,5,6,7,8,9,10,11,37,38,39]. These dislocations glide in the slip planes intersecting the surface. The lines observed are the bottoms of dislocation half-loops. The dark points seen around the indentation are grown-in threading dislocations. Moreover, dislocation loops gliding in the basal {0001} plane parallel to the surface can be also observed (indicated by the red arrows in Figure 1a). These dislocations are curved and do not correspond to any crystallographic direction, which may indicate low values of the Peierls barrier for dislocations gliding in this slip plane, because segments of dislocations introduced at low temperatures into covalent crystals are usually located in the Peierls potential valleys. It can be seen that in sample S1, the basal plane dislocations move over larger distances from the imprint center than those in planes intersecting the surface, a finding which correlates well with the results of molecular dynamics simulations [43], which predicted that dislocations are most mobile on the basal plane and least mobile on the {10$\bar{1}$1} pyramidal plane. However, in Figure 1b, in which the imprint in the S5 sample is shown, only dark lines elongated in the <11 $\bar{2}$ 0> directions can be seen (curved dislocation lines are grown-in dislocations in the wings [58]). As the dislocation density above the slit in this sample is approximately 10^8^ cm^−2^ and a large number of dislocations exist at the wing merger boundaries, such dislocations can play a role as effective obstacles to basal plane dislocations and prevent their expansion.

**Figure 2:**
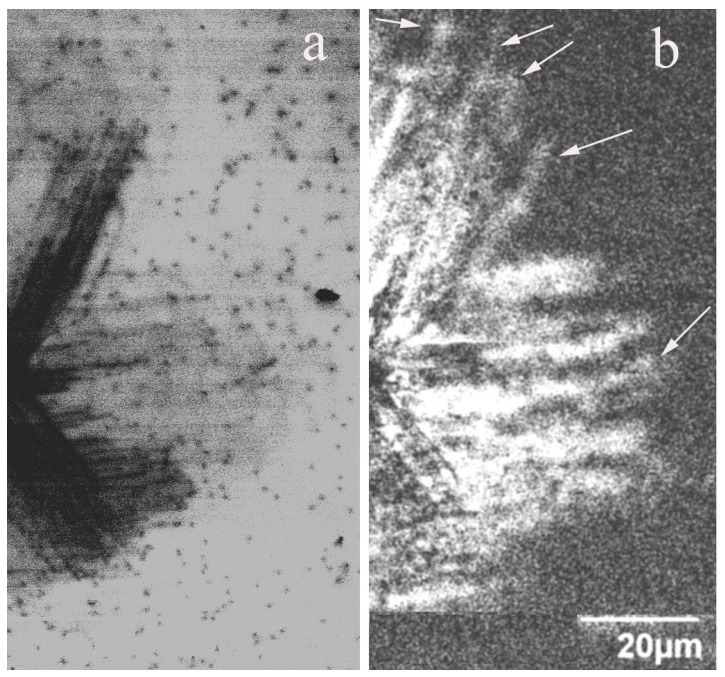
Fragments of dislocation rosette CL images of the sample S1, obtained at 365 nm (**a**) and at 400 nm (**b**). *E_b_* = 10 keV. Arrows indicate bright lines longer than dark lines.

Figure 2 shows the CL images obtained for the same area of the dislocation rosette at wavelengths of 365 nm (near-bandgap emission) and 400 nm (DRL). It is seen that some bright lines in Figure 2b, corresponding to DRL (shown with arrows), are noticeably longer than the dark lines in Figure 2a, corresponding to dislocations with a high recombination rate. Since the slip planes intersecting the surface were assumed to be {10 $\bar{1}$ 1} pyramidal [1,3,4] or {10 $\bar{1}$ 0} prismatic planes [38,39], it can be assumed that the bright lines are associated with dislocations gliding in the slip planes, differing from the slip planes of dislocations without DRL. Additionally, images of bright lines are usually wider than those of dark lines. This seems to confirm the assumption that the DRL is associated with dislocation trails forming behind dislocations gliding in the pyramidal slip planes inclined towards the surface. Indeed, in this case, the projection of the trails should be wider than the dislocation image itself. It should also be noted that defects with the DRL were observed in samples S1, S5 and S6 but not in samples S2–S4.

**Figure 3:**
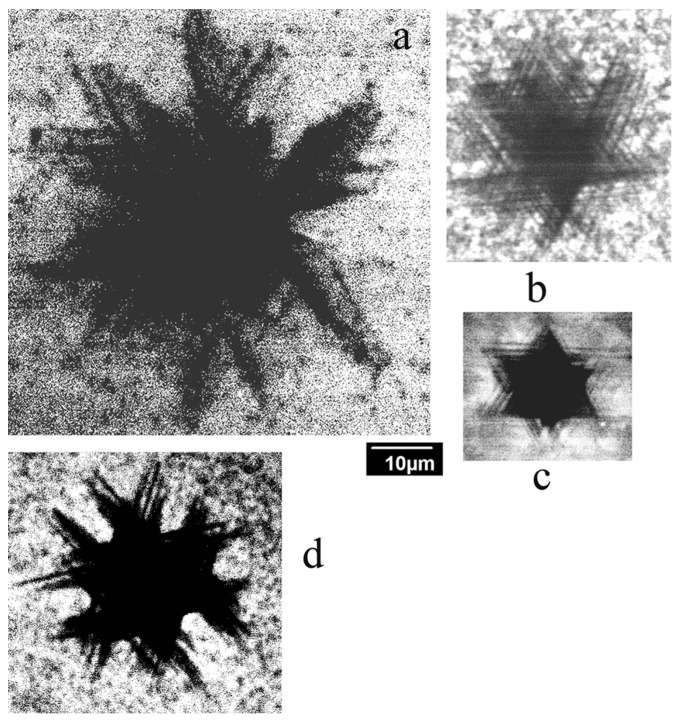
CL images of dislocation rosettes formed at a load of 1 N at room temperature in samples S1 (**a**), S2 (**b**), S4 (**c**) and S3 (**d**). *E_b_* = 10 keV.

As the transition from the double-kink mechanism of dislocation motion to the overcoming of localized obstacles should depend on the density and strength of the obstacles [49], it could be expected that the dislocation velocity and, as follows from Equation (3), travelling distance of dislocations can vary significantly from crystal to crystal, depending on the density and/or strength of the obstacles. CL images of dislocations introduced at a load of 1 N into the samples S1–S4 are shown in Figure 3. It can be seen that despite the use of the same load and similar dopant concentrations in the S1–S3 samples, the rosette size differs by several times. Moreover, while in samples S1 and S3, in which the dislocation travelling distances are larger than those in the other samples, dislocation arms running from the corners of the imprint can easily be seen, in samples S2 and S4, the star-of-David-like dislocation rosettes are formed due to cross slip (a shift of a screw dislocation from the primary slip plane to another one), even at room-temperature deformation. In comparison, in the S3 sample, the star-of-David-like dislocation rosettes were observed only after deformation at T > 573 K [8].

As follows from Equation (3), the dislocation travelling distance depends on the material parameters, such as *A*, τ_0_, *V*_0_ and *m*, at a power smaller than 1 and on the load. As the load was the same for all the studied samples, it is difficult to explain the difference in the rosette size in the framework of the double-kink mechanism of dislocation motion.

**Figure 4:**
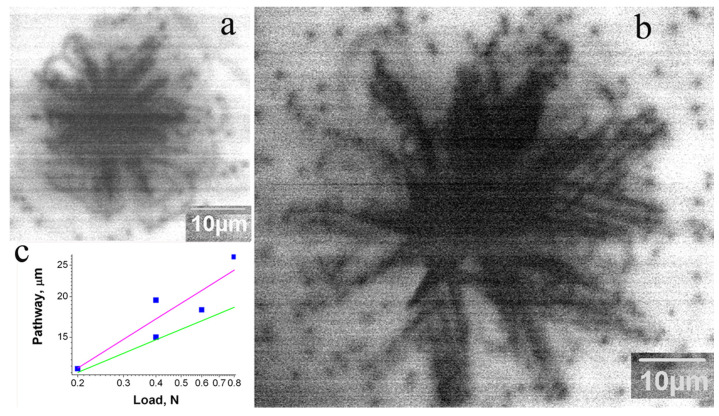
Monochromatic CL images of dislocation rosettes in the sample S1 produced at 365 nm with loads of 0.2 (**a**) and 0.8 N (**b**). (**c**) The measured dependence of dislocation travelling distance on the load (blue squares). Lines show the dependencies *L*~*P*^0.5^ (magenta) and *L*~*P*^0.5^ (green).

**Figure 5:**
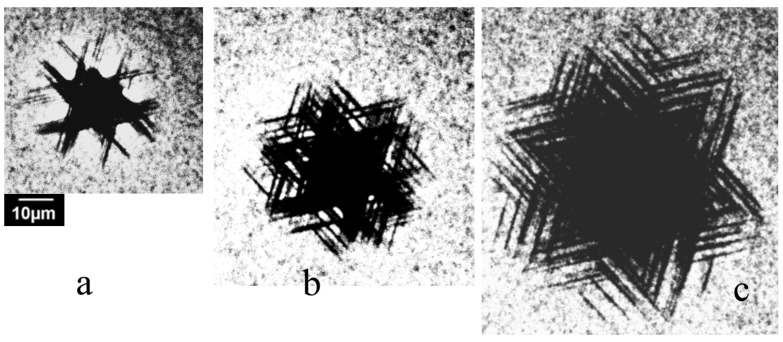
Panchromatic CL images of dislocation rosettes in the sample S3 introduced under a load of 1 N at room temperature (**a**) and at 600 °C (**c**). The rosette obtained via deformation at room temperature and then annealed at 600 °C is shown in (**b**).

A study of the dislocation rosette size’s dependence on the load shows (Figure 4) that the rosette form is practically independent of the load *P*, and only the size increases with *P*. As follows from Equation (3), *L* is proportional to *P^m^*^/(2*m*+1)^, and *m*/(2*m* + 1) changes from 1/3 for *m* = 1 to ~1/2 for large *m* values. Due to this small difference, it is difficult to experimentally obtain an *m* value with suitable precision, especially for a large *m*. However, as seen in Figure 4c, the dependence of *L* on *P* is closer to *P*^0.5^ (magenta line) than to *P*^1/3^ (green line). This means that *m* is rather large (3 ≤ *m* ≤ 5, as estimated in [8]) and correlates well with the simulation results in [43,65]. An increase in deformation or annealing temperature also affects the rosette form (Figure 5). At sufficiently high temperatures, star-of-David-like dislocation rosettes formed in all the samples studied, which correlates well with the results of [2,4,36,60]. It seems that the smaller the size of rosettes introduced at room temperature is, the lower temperature at which the cross slip mechanism becomes effective will be.

**Figure 6:**
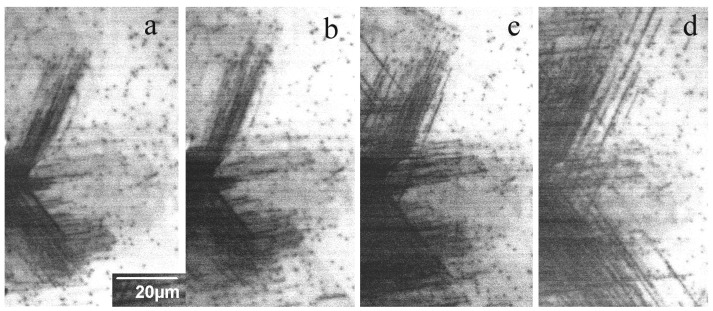
Fragment of dislocation rosette in the sample S1 measured at 365 nm after deformation (**a**) and subsequent annealing at 200 (**b**), 300 (**c**) and 400 °C (**d**) for 10 min. *E_b_* = 10 keV.

**Figure 7:**
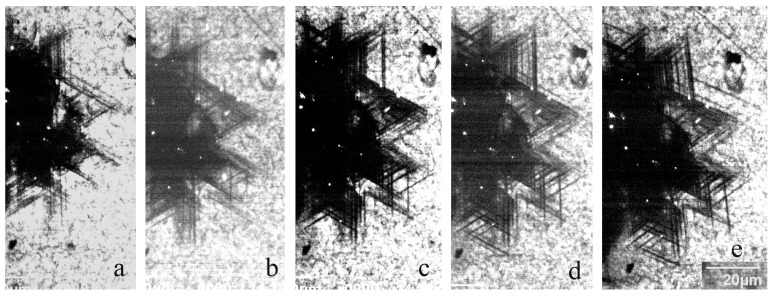
Fragment of a dislocation rosette in the sample S6 measured in the panchromatic mode after deformation (**a**) and subsequent annealing at 200 (**b**), 300 (**c**), 400 (**d**) and 500 °C (**e**) for 10 min. *E_b_* = 10 keV.

Typical modifications of the dislocation rosette formed due to annealing in the samples S1 and S6 are shown in Figure 6 and Figure 7, respectively. It can be seen that even in the sample closest to perfection, S1, the leading dislocations essentially do not increase to 200 °C, although the dislocation density in the highly stressed region increases. Annealing at 300 °C further leads to a pronounced increase in the dislocation number, but the travelling distance from the imprint center essentially does not change. An increase in the travelling distance occurs only when the annealing temperature increases above 350 °C. In the sample S6, grown using the same method with nearly the same donor concentration but a higher grown-in dislocation density, the dislocation travelling distance is almost unchanged up to 500 °C, although the number of dislocations and cross slip events is noticeably increased (Figure 7). The annealing effect on the defect structure in other samples is qualitatively very similar. The main difference lies in the significant change in the dislocation travelling distance and the temperature at which cross slip starts to operate. These results clearly demonstrate some extrinsic effect on the dislocation travelling distance.

**Figure 8:**
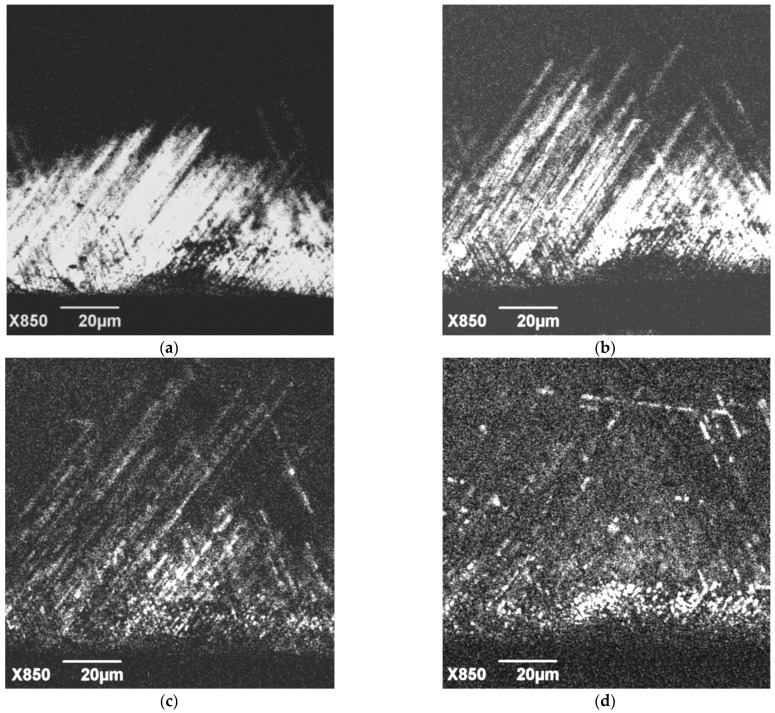
Monochromatic CL images of dislocations generated from a scratch in the sample S1 at 400 nm and room temperature obtained after deformation (**a**) and after subsequent annealing at 200 °C (**b**), 300 °C (**c**) and 400 °C (**d**).

The annealing effect on the defects demonstrating the DRL is more pronounced. As seen in Figure 8a, the length of these defects slightly increases after annealing at 200 °C (Figure 8b), while their brightness essentially decreases. If the annealing temperature increases to 300 and 400 °C, these defects practically disappear, although a few new defects arising due to annealing can be seen in Figure 8c,d. It should be noted that a decrease in the CL emission intensity of these defects due to annealing was also observed in [39], and their disappearance after annealing at 500 °C was observed in [60].

**Figure 9:**
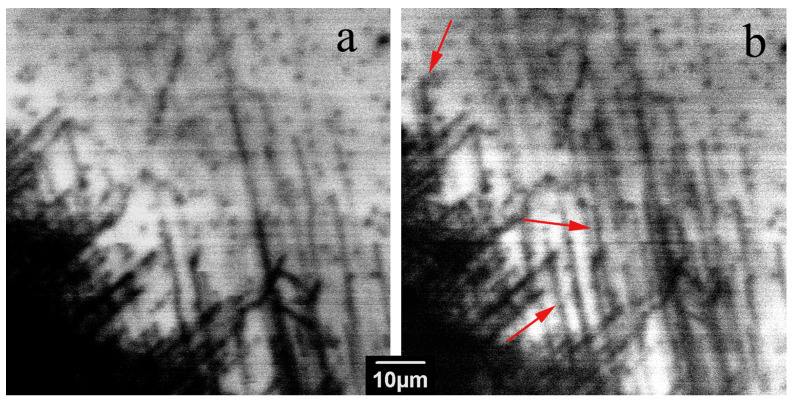
Panchromatic CL images of dislocations generated from scratches obtained at liquid nitrogen temperature in the 1st (**a**) and 5th frame scan (**b**). *E_b_* = 10 keV. The shifted dislocations are shown with red arrows.

A study of the LEEBI effect on the dislocation structure in all the samples studied shows that, similar to previous investigations [7,11,54,55], only a few dislocations are shifted due to LEEBI, although, as seen in Figure 9, a few of them can move even at liquid nitrogen temperature (shown with red arrows). The images shown in Figure 9 were obtained in the first and fifth frame scans with a frame scan time of 160 s/f (seconds per frame). At room-temperature irradiation, the number of mobile dislocations did not noticeably increase. Since this number is rather small and unpredictable, it is difficult to infer the temperature dependence of the dislocation velocity; however, the ability of dislocations to move at liquid nitrogen temperature under LEEBI assumes that the activation energy for dislocation glide is not higher than 10–20 meV. It is also observed that LEEBI leads to the disappearance of DRL, which correlates well with the previously obtained results [7,36,60].

## 4. Discussion

In a typical case, both thermal annealing and LEEBI stimulate dislocation motion. Temperature stimulates the overcoming of the barrier; thus, under applied stress, dislocation mobility should increase with temperature. In the case of annealing, dislocation motion is driven by the residual stress stored due to limited dislocation mobility at lower temperatures. It is widely accepted that in covalent semiconductors, the overcoming of the Peierls barrier is achieved through two successive processes: the formation of a double kink on a straight dislocation line and the subsequent migration of the generated kinks along the dislocation [12,13]. As a consequence, the energy governing the dislocation motion consists of two terms: double-kink formation energy and kink migration energy. Under LEEBI, the energy released during the recombination of excess carriers generated by the e-beam at dislocation-related centers is redirected to lower the barrier for the dislocation glide [67]. The two barriers or the barrier for kink formation alone can be lowered. It should also be taken into account that the Peierls barrier values depend on the dislocation type and the acting slip plane. However, for the same dislocation type, the dislocation behavior under annealing should be similar. In the present study, it was shown that the dislocation travelling distance changes from crystal to crystal, while most of the dislocations remain immobile. This can easily be explained by the obstacle effect on the dislocation motion, taking into account the fact that the procedure, annealing and LEEBI can all stimulate dislocation release from weak obstacles.

The results presented show that, in accordance with the previously obtained results [1,3,4,38,39], at least two slip planes are active in GaN at room temperature: the basal plane and the prismatic or pyramidal plane intersecting the surface. A comparison of dislocations producing DRL and dark CL contrast shows that one cannot exclude the possibility that two different slip planes intersecting the surface are active at room temperature. They could be {10 $\bar{1}$ 1} [1,3,4,68] and {11 $\bar{2}$ 2} [68,69] pyramidal planes or {10 $\bar{1}$ 0} prismatic planes [38,39]. In any case, the wider DRL image indicates that this emission corresponds not to the dislocations themselves but to dislocations gliding in the pyramidal planes and generating point defects during their motion. This statement is supported by the low thermal stability of these defects and their disappearance under LEEBI. Of course, the disappearance of DRL defects could be explained by dislocation movement to depths greater than the electron beam penetration depth. However, if DRL is associated with screw dislocations dissociated in the basal plane, as assumed in [6,38,39], in order to move deeper along the slip plane inclined to the surface, the stacking fault between two partials should be constricted. Additionally, it should be noted that despite the rather similar dislocation distributions around the imprints, DRL was observed only in three of the samples studied, which also indicates that DRL is not related to the dislocations themselves. Indeed, if the defects formed in the slip planes are complexes of intrinsic point defects with impurities, their concentration and type should depend on the impurity content.

Figure 3 shows that the dislocation rosette size essentially changes from crystal to crystal. As seen in Figure 4c, the dislocation rosette size increases approximately in line with *P*^0.5^. A similar dependence of the rosette size on *P* can be determined from the data presented in [1]. This allows us to compare the rosette sizes measured in the present work with those from numerous previous works formed at the different loads. Such a comparison shows that the difference between different crystals can be even larger than that shown in Figure 3. The dopant concentrations in the n-type samples used in the present work are very similar; moreover, they are lower than the values at which the dependence of dislocation mobility on the dopant concentration is usually observed (higher than 10^18^–10^19^ cm^−3^) [52]. This supports the assumption that dislocation mobility in GaN is determined not only by intrinsic mechanisms but also by extrinsic ones. It should be mentioned that, as observed in [70], the dimensions of dislocation structures induced via scratching are proportional to the scratch width, which allows the authors to propose the simple method for the estimation of the maximum thickness of the layer damaged under wafer polishing by measuring the scratch width. Unfortunately, the results obtained in the present paper show that the thickness of the damaged layer can depend on the GaN’s state of perfection. Thus, such a simple approach to the estimation of the damaged layer thickness is hardly applicable to GaN. In samples with a higher obstacle density, the dislocations need higher stress for glide in the slip plane in order to overcome the obstacles. It is probable that such stress stimulates cross slip, which allows us to explain the correlation of temperature with the formation of star-of-David-like dislocation rosettes and the rosette size. Indeed, if obstacles block dislocation glide in the slip plane, the dislocations will try to overcome them via cross slip.

Relatively high dislocation mobility at room temperature indicates that the Peierls barrier in GaN does not exceed 1 eV, which correlates well with the 0.54–0.66 eV values obtained through molecular dynamics simulations [43] and 0.45–0.65 eV estimated from the temperature dependence of the dislocation rosette size [8,10]. It should be noted that such values are a few times lower than those for other semiconducting covalent crystals, such as Si, SiC and GaAs. Moreover, under LEEBI, some dislocations can glide even at liquid nitrogen temperature, which allows us to estimate the activation energy for REDG as not exceeding a few tens of meV. If the Peierls barrier is low, as seems to take place in GaN, some additional mechanisms may essentially affect the mobility, and indeed, the dislocation motion can be determined, for example, by overcoming the obstacles, the type and concentration of which can essentially change from crystal to crystal. This assumption seems to be supported by the fact that even in the HVPE crystals with the largest rosette size, which implies the smallest obstacle effect, only a small number of dislocations are shifted under LEEBI (see, e.g., Figure 9). A similar behavior was previously observed in [7,11,38,54]. As observed in [60], only a few dislocations propagated away from the center of indentation due to annealing at 500 °C, which allows one to assume that other dislocations are pinned by obstacles. Under e-beam irradiation, the dislocation motion is jerky, which also supports the assumption that dislocations jump from one obstacle to other one.

A comparison of Figure 1a,b shows that while, in the S1 sample, the travelling distance of the basal dislocations is larger than that of dislocation gliding in the other slip planes, in the S5 sample, no basal dislocations are revealed, which allows us to assume that threading dislocations play the role of effective obstacles for basal plane dislocations. For the slip planes intersecting the surface, threading dislocations are not so effective, and other types of obstacles, which can be associated with precipitates, are more effective. Dislocations can also attract impurities due to the hydrostatic stress field associated with their formation of impure atmospheres. While moving, the dislocations are shifted away from these atmospheres, which are dragged behind. Such atmospheres can also pin dislocations, especially at low stress, as observed in Si [71].

**Figure 10:**
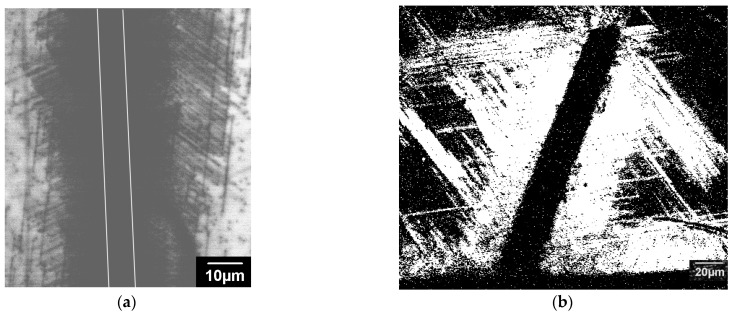
Monochromatic CL images of dislocations generated from a scratch in the sample S1 at 365 nm (**a**) and 400 nm (**b**) and room temperature. The dark band in (**b**) corresponds to the scratch, and in (**a**) the scratch is marked by white lines.

Thus, it seems that in GaN, two mechanisms control the dislocation dynamics, i.e., overcoming the Peierls barrier and overcoming localized obstacles, which work simultaneously. The observed difference in the dislocation travelling distances in the studied samples is determined by the number and strength of the obstacles. As these two mechanisms have a different stress dependence and the shear stress decays essentially in line with the distance from the imprint or scratch, a boundary between the areas where each is more effective may exist. Indeed, as seen in Figure 10, the dislocated regions formed near scratches can be roughly divided into two parts. The region adjacent to the scratch contains a high density of dislocations with approximately the same travelling distances. At larger distances from the scratch, the density of dislocations decreases, while their travelling distances varies in a wide range. A similar behavior, albeit less obvious, was also observed near the imprints. The high stress stimulates the overcoming of obstacles; thus, in the close vicinity of scratches or indentations, the dislocation mobility should be controlled by the double-kink mechanism [49], which makes it easy to explain such images. Indeed, the double-kink mechanism assumes that the dislocation mobility is mainly determined by the overcoming of the Peierls barrier, which is practically the same for all dislocations and all GaN samples. However, when the stress is not high enough to overcome obstacles, the travelling distance will depend on the obstacle number and strength. As the obstacles are randomly distributed throughout the crystal, the travelling distances of individual dislocations can essentially differ. Annealing, as used in the present study, or e-beam irradiation seems to only assist in dislocation release from weak obstacles. This allows us to explain why such treatments stimulate the shift of only a small number of dislocations. Dislocations move until they meet a strong obstacle, and the distance covered by the dislocations allows us to estimate that the distance between such obstacles is approximately 10 μm.

## 5. Conclusions

Thus, it is shown that the Peierls barrier for dislocation glide in GaN is essentially lower than that in the common electronic semiconductors, such as Si, SiC and GaAs. The travelling distance of dislocations in a few GaN layers grown through the HVPE, MOCVD and ELOG methods and possessing different dislocation densities is shown to essentially differ. This clearly indicates that the mobility of a dislocation in the state-of-the-art GaN is not entirely determined by its intrinsic properties; the obstacle effect can play the essential role. Results confirming the essential obstacle effect on dislocation motion in GaN were presented and discussed. It is shown that threading dislocations play the role of effective obstacles for basal plane dislocations. It is shown that under LEEBI, the activation energy for the dislocation glide decreases to a few tens of meV. Therefore, under e-beam irradiation, the dislocation movement is controlled by overcoming localized obstacles. The results obtained could be useful for the prediction of the effects of loading during processing or device operation on dislocation generation and multiplication.

## Data Availability

The data that support the findings of this study are available from the corresponding author upon reasonable request.
